# Serum Vitamin A in Relation to Genomic Biomarkers in Children: A Prospective Study

**DOI:** 10.3390/nu18203313

**Published:** 2026-10-09

**Authors:** Yan Wang, Kuiqing Huang, Chen Liu, Yi Wang, Jinghan Jiang, Zhijian Chen, Ronghua Zhang, Peiwei Xu, Xiaoming Lou

**Affiliations:** 1 Zhejiang Provincial Center for Disease Control and Prevention, Hangzhou 310051, Chinaxmlou@cdc.zj.cn (X.L.); 2 Yiwu Center for Disease Control and Prevention, Yiwu 322000, China; 3 School of Public Health, Hangzhou Medical College, Hangzhou 310059, China

**Keywords:** vitamin A, telomere length, ribosomal DNA copy number, mitochondrial DNA copy number, genomic stability, children

## Abstract

Background/Objectives: Vitamin A (VA), which is fat-soluble, belongs to the group of carotenoids, a class of highly effective antioxidant and genoprotective compounds (including astaxanthine, zeaxanthine, luteine). It plays a critical role in cellular growth, differentiation, and oxidative stress regulation, but prospective evidence linking childhood VA to subsequent genomic stability is scarce. This study investigated whether baseline VA levels were associated with later measurements of relative telomere length (RTL), four ribosomal DNA copy numbers (rDNA-CN: 5S, 18S, 28S, and 45S), and mitochondrial DNA copy number (mtDNA-CN) in children over a 4-year follow-up. Methods: Baseline VA levels were measured in 2021. In 2025, peripheral blood genomic DNA was extracted, and RTL, rDNA-CNs, and mtDNA-CN were quantified using qPCR. Multivariable linear regression and restricted cubic spline regression (RCS) were employed to examine the associations and dose–response shapes. Sex-stratified analyses with interaction tests were performed to evaluate effect modification by sex. Results: Among 215 children, baseline VA was positively associated with RTL, 5S rDNA-CN, 18S rDNA-CN, and 45S rDNA-CN after covariate adjustment. RCS analyses revealed strictly monotonic positive dose–response curves between VA and RTL, 5S rDNA-CN, and 18S rDNA-CN, with no statistically significant sex interaction (all *p* for interaction > 0.05). Conclusions: Higher childhood VA levels are positively associated with longer telomeres and higher rDNA-CN in later childhood, suggesting that adequate early-life VA may confer long-term benefits for ribosomal genomic stability and cellular aging. These observations expand our understanding of nutrition-related modulators of child genomic homeostasis and support further investigation into nutritional interventions targeting genomic protection in early life.

## 1. Introduction

The repetitive/multi-copy genomic features, such as telomeres, mitochondrial DNA (mtDNA), and ribosomal DNA (rDNA), play critical roles in maintaining genome stability [1,2,3], regulating cellular senescence [1,2,4], and influencing trait variance in humans [5]. Telomeres, the DNA–protein structures at the end of chromosomes, are maintained around an equilibrium distribution, and failure to maintain the length distribution could predispose individuals to age-related diseases or cancer [6]. MtDNA copy number (mtDNA-CN) is considered a proxy for mitochondrial function [7], with alterations in the mtDNA content indicating mitochondrial damage in population studies of cardiovascular diseases or cancer [8,9,10]. To meet the demands of ribosome biogenesis, two multicopy rDNA arrays, 45S and 5S, encode the mature rRNA components of ribosomes. The 45S rDNA loci consist of sections encoding the 5.8S, 18S, and 28S rRNAs [11]. The copy number of rDNA (rDNA-CN) generally remains within a particular range across many organisms [12]. Perturbations in rDNA-CN result in genome instability and might promote carcinogenesis [13]. Relative telomere length (RTL), mtDNA-CN, and rDNA-CN are highly sensitive to inflammation and oxidative stress, serving as promising molecular biomarkers reflecting genomic homeostasis [14,15,16].

Fat-soluble vitamins, including vitamin A, are energy-free molecules that are essential to the body’s functioning and life [17]. The main biologically active forms of vitamin A are retinol (C_20_H_30_O) and retinoic acid (RA) (C_20_H_28_O_2_). Vitamin A possesses antioxidant and antigenotoxic properties [18,19], with the ability to alleviate oxidative stress-induced DNA damage [19]. Vitamin A derivatives could exert anti-cancer effects by inducing cancer cell differentiation, cell-cycle arrest, apoptosis, and inhibiting invasion and inflammation [20]. Carotenoids represent a large family of plant-derived lipophilic pigments. Some carotenoids (e.g., β-carotene, C_40_H_56_) act as provitamin A [19]. Similar to fat-soluble vitamins, carotenoids possess potent antioxidant capacity to reduce lipid peroxidation as well as oxidative stress [21]. Accordingly, these compounds also exhibit antigenotoxic activity [19]. Carotenoids can regulate adipocyte differentiation and reduce fat accumulation, modulate apoptosis-related signals in epithelial cells, and exert antioxidant and anti-inflammatory protective effects on liver, retinal and immune cells [21]. Carotenoids exert their functions on germ cells primarily via their active metabolite, RA [22]. RA could mediate spermatogonial differentiation and participates in multiple steps of spermatogenesis [23]. By mitigating oxidative-mediated genomic insults, vitamin A may profoundly affect the copy number variation in these repetitive genomic elements and genomic stability regulation [24].

Mitochondrial dysfunction severely disrupts the function of high-energy-demanding organs, including the brain, heart, liver, and skeletal muscle, with impaired growth being one of its most prominent adverse outcomes [25]. Lower rDNA-CN has consistently been associated with higher body weight and body mass index (BMI) [26], and short telomeres could contribute to the development of obesity and metabolic disorders very early in life, which can have a major impact on health [27]. Fat-soluble vitamins are involved in the regulation of genomic stability [19,28]. Metabolites of vitamin D_3_ can activate the transcription factor vitamin D receptor (VDR), which is expressed in the vast majority of human tissues and cell types. Through VDR, vitamin D modulates the epigenome of various tissue cells, regulates numerous physiological functions, including metabolism, bone formation, and immune responses [29], and targets DNA-repair pathways to attenuate cellular DNA damage [28]. As another essential member of fat-soluble vitamins, vitamin A and its active metabolites might also be tightly involved in mitochondrial function and genomic maintenance. Vitamin A and its derivatives can enhance mitochondrial activity [30] and regulate the expression of specific mitochondrial genes [31]. In vitro cellular experiments revealed that 9-cis RA increases the RNA expression of NADH-dehydrogenase subunit 1 (*ND1*) and cytochrome c oxidase subunit I (*COX1*) [31]. At physiologically appropriate doses, RA might exert genome-protective effects, whereas excessive supplementation might induce pro-oxidant effects [19]. However, existing relevant research is largely limited to cross-sectional investigations focusing merely on vitamin A and telomere length [32]; prospective evidence clarifying the associations between vitamin A nutritional status and RTL, mtDNA-CN as well as rDNA-CN remains scarce, especially among children who are in critical developmental stages with high nutritional susceptibility. In addition, established lifestyle and anthropometric factors are closely implicated in genomic stability. Obesity and elevated BMI contribute to increased genomic instability, telomere attrition, and mitochondrial dysfunction by promoting chronic oxidative stress and inflammatory responses [33,34]. Similarly, unhealthy lifestyle exposures, including smoking, can aggravate DNA damage and disrupt the stability of the genome [35]. Therefore, these confounding variables were fully considered and adjusted for in the present study.

Given the critical roles of RTL, mtDNA-CN, and rDNA-CN in maintaining genome stability, cellular senescence, and disease progression, as well as the antioxidant and genomic protective properties of vitamin A, we hypothesized that serum vitamin A levels are significantly associated with the multi-copy genomic elements. We adopted a prospective observational cohort design to test this hypothesis. Serum vitamin A was determined at the baseline survey in 2021, while RTL, mtDNA-CN and rDNA-CN were quantified at the follow-up survey in 2025. Specifically, we aimed to examine the independent relationships between vitamin A and RTL, mtDNA-CN, and rDNA-CN in general children after accounting for relevant covariates to explore whether vitamin A may serve as a potential risk or protective factor for these genomic biomarkers, and to assess whether such associations differ by sex. The findings of this study aim to provide novel epidemiological evidence for elucidating the nutritional regulatory factors of genomic stability and offer new insights for the early prevention of aging-related lesions and genomic dysfunction in pediatric populations.

## 2. Materials and Methods

### 2.1. Study Population

**Figure 1:**
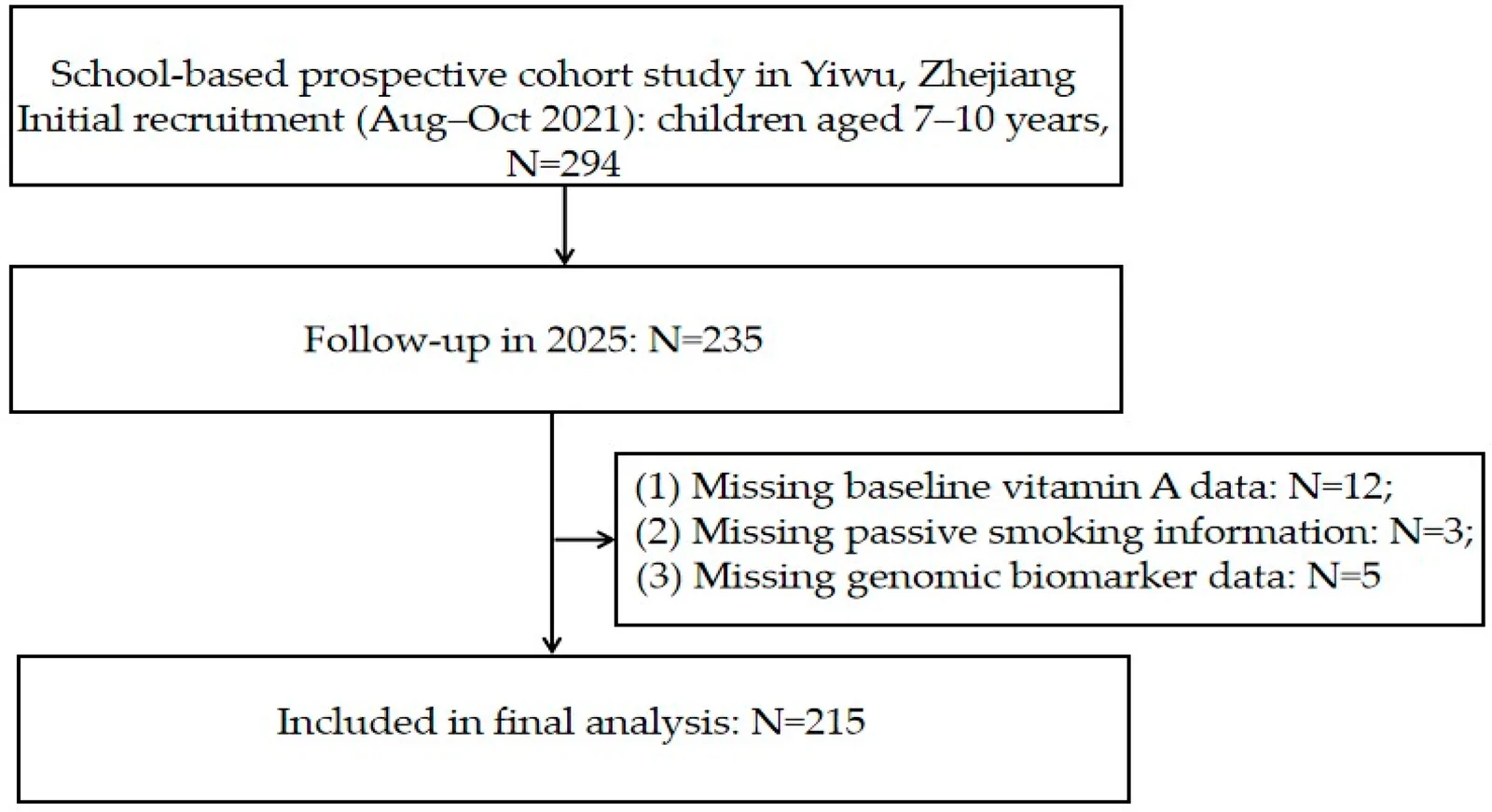
Flowchart of study participants.

This study was performed using data from a school-based prospective cohort study that was established in Yiwu, Zhejiang Province, China. The cohort initially recruited 294 healthy children aged 7–10 years from a primary school in Yiwu City from August to October 2021 [36]. The school was selected based on convenience sampling. The school administration provided approval to carry out the survey and specimen collection. Participants were re-examined in 2025, with 235 children successfully followed up. The inclusion criteria were as follows: (1) children who participated in the 2021 baseline survey of the Yiwu school-based cohort; (2) those who completed follow-up re-examination in 2025; and (3) participants with complete baseline vitamin A measurement, complete baseline covariates (age, sex, BMI, passive smoking exposure), and valid genomic biomarker data collected in 2025. The exclusion criteria were as follows: (1) children with missing data of vitamin A; (2) children without passive smoking information; and (3) children with missing genomic biomarker data. The flowchart was presented in Figure 1. After excluding 20 participants with missing data, a total of 215 eligible children from the Yiwu cohort were included in the final analysis.

Written informed consent was obtained from all guardians prior to sample collection. This study was approved by the Ethics and Human Subject Committee of the Zhejiang Provincial Center for Disease Control and Prevention (Approval No. 2020-040-01, 2025-022-01).

### 2.2. Data Collection

Baseline information about the participants was obtained through face-to-face interviews by well-trained staff using a standardized questionnaire, with parents or guardians completing the questionnaire for participants. The demographic variables included location of residence, sex, and age. Passive smoking was defined as the presence of any family members or coworkers who frequently smoke in their vicinity (yes or no). Weight and height were measured to the nearest 0.1 kg and 0.1 cm, and body mass index (kg/m^2^) was calculated by dividing weight (kg) by the square of height (m^2^). Samples of fasting blood were collected at baseline and follow-up surveys using standardized protocols. All blood specimens were centrifuged at 3000 rpm for 15 min at 4 °C to separate serum. The serum aliquots were transferred into 1.5 mL polypropylene tubes and preserved at −80 °C pending laboratory testing. Baseline serum specimens were collected and tested for vitamin A concentrations in 2021, whereas whole blood samples obtained during follow-up were used for the quantification of gene copy numbers. This study was reported following the Strengthening the Reporting of Observational Studies in Epidemiology (STROBE) guidelines. The completed STROBE checklist is provided as Supplementary Materials (Table S1).

### 2.3. Measurement of Vitamin A

Baseline serum vitamin A concentrations were determined using liquid chromatography–tandem mass spectrometry (LC-MS/MS) with the Triple Quad 4500 system (AB SCIEX, Framingham, MA, USA). More details can be found in Supplementary Materials and Table S2.

### 2.4. Measurement of Genomic Biomarkers

RTL, 5S rDNA-CN, 5.8S rDNA-CN, 18S rDNA-CN, 28S rDNA-CN, 45S rDNA-CN, and mtDNA-CN are genomic biomarkers. These genomic biomarkers were used as primary outcomes in this study. These biomarkers were selected for three reasons: (1) they are repetitive multi-copy genomic features highly responsive to oxidative stress and inflammation, and can mirror genomic homeostasis [1,2,3,14,15,16]; (2) high-quality sequencing data for these markers were accessible for all included participants; (3) In human epidemiological studies, they can be used to characterize cellular senescence, mitochondrial dysfunction and genomic instability [37].

Genomic DNA was extracted using a HiPure Blood DNA Mini Kit (D3111, Magen Biotechnology, Guangzhou, China). Briefly, the sample was transferred into a 1.5 mL centrifuge tube, mixed with 220 µL of Buffer ATL and 20 µL of Proteinase K by vortexing, and incubated at 55 °C for 1–3 h until complete digestion and clarification with several inversions during incubation. Then, 10 µL of RNase A was added to the digest, mixed by inversion, and kept at room temperature for 10 min. Subsequently, 250 µL of Buffer DL was added, followed by high-speed vortexing for 10 s and incubation in a 70 °C water bath for 10 min. After centrifugation at 12,000× *g* for 3 min, the supernatant was transferred to a new 1.5 mL centrifuge tube, mixed with 250 µL of absolute ethanol by vortexing for 15 s. The mixture including precipitate was loaded onto the spin column assembled in a 2 mL collection tube and centrifuged at 12,000× *g* for 1 min; the sample was loaded in portions if the volume exceeded 750 µL, and 14,000× *g* centrifugation for 3–5 min was applied when column clogging occurred. The flow-through was discarded, and the column was washed sequentially with 500 µL of Buffer GW1 and 650 µL of Buffer GW2, each followed by centrifugation at 12,000× *g* for 1 min. After the final wash, the column was centrifuged at 12,000× *g* for 2 min to remove residual liquid. The column was transferred to a new 1.5 mL centrifuge tube, 70 µL of Buffer AE preheated to 70 °C was added onto the center of the column membrane, and the tube was kept stationary for 3 min before centrifugation at 12,000× *g* for 1 min. Finally, the DNA-binding column was discarded; the obtained DNA was stored at 2–8 °C for short-term use and at −20 °C for long-term preservation. DNA integrity was examined via 1% gel electrophoresis, and DNA purity was determined using a Micro Drop SE spectrophotometer (BIO-DL, Shanghai, China), with acceptable OD260/280 values between 1.6 and 2.0.

Quantitative real-time PCR (qPCR) was performed to detect target genes using an ABI ViiA 7 real-time PCR system (Applied Biosystems, Waltham, MA, USA). Briefly, SYBR Green Master Mix (TSE203, TSINGKE, Beijing, China) was thawed at 4 °C, mixed gently by inversion, and briefly centrifuged. A 20 μL reaction system was prepared on ice, containing 10 μL SYBR Green Master Mix, 0.4 μL forward primer (10 μM), 0.4 μL reverse primer (10 μM), and 20 ng template DNA, with RNase-free water added to a final volume of 20 μL. The reaction mixture was briefly centrifuged to ensure all liquid was collected at the bottom of the wells. The amplification procedure was set as follows: initial predenaturation at 95 °C for 5 min, followed by 40 cycles of 95 °C for 10 s and 60 °C for 30 s, and fluorescence signals were collected at the end of each cycle. All samples were analyzed in triplicate on the same 384-well plate. Tables S3–S5 presented the Primers for ribosomal DNA copy number, mitochondrial DNA copy number, and relative telomere length.

The rDNA-CN was normalized to the *TP53* gene, whereas mtDNA-CN (based on the mitochondrial gene *ND1*) and RTL were normalized to the β-globin gene. The relative levels of rDNA-CN, mtDNA-CN, and RTL were calculated using the 2^−△Ct^ method.

### 2.5. Statistical Analysis

The characteristics of the participants are presented as the mean ± standard deviation (SD) for continuous variables, while categorical variables are described using frequencies and proportions. Concentrations of vitamin A and copy numbers of genomic biomarkers (rDNA-CNs, mtDNA-CN, and RTL) were natural logarithmically (ln) transformed to enhance data normality and minimize the impact of potential outliers.

The associations between vitamin A and genomic biomarker were investigated by fitting linear regressions, adjusting for potential confounders, including age, sex, BMI, and passive smoke. In addition, the dose–response associations of vitamin A with levels of genomic biomarkers were assessed by restricted cubic spline (RCS) regression with 3 knots (P5, P50, and P95). Stratified analysis was conducted to examine whether the associations between vitamin A and levels of genomic biomarkers were modified by sex. To facilitate the interpretation of the associations, we converted the regression coefficient β and its corresponding 95% confidence interval (95% CI) to percent changes (%Δ) in genomic biomarkers with an IQR increase in vitamin A concentrations. Multiple testing was corrected using the Benjamini–Hochberg false discovery rate (FDR) procedure. As for the degrees of freedom for each test, first, for the multivariable linear regression model, the independent variables included vitamin A, age, sex, BMI, and passive smoking; thus, the number of independent variables in the model was k = 5. Let n denote the total sample size. For the overall F-test of the regression model, the numerator degrees of freedom were df1 = k = 5, and the denominator degrees of freedom were df2 = n − k − 1 = n − 6. The degrees of freedom for the *t*-tests of each regression coefficient were also df = n − k − 1 = n − 6. Second, restricted cubic spline regression was fitted with three knots (P5, P50, and P95), and the spline term was specified with 2 degrees of freedom. Finally, for sex-stratified linear regression, linear regression models were fitted separately in males and females. Since sex was invariant within each stratum, it was not included as a covariate in the stratified models. Each stratum included four independent variables: vitamin A, age, BMI, and passive smoking (k = 4). The residual degrees of freedom were df_male = n_male − k − 1 = n_male − 5 for the male stratum and df_female = n_female − k − 1 = n_female − 5 for the female stratum, where n_male and n_female denote the sample sizes of males and females, respectively.

All analyses were performed with R software (version 4.4.2; R Development Core Team). A *p* < 0.05 was considered statistically significant.

## 3. Results

### 3.1. Basic Characteristics of Participants

Table 1 showed the demographic characteristics and levels of genomic biomarkers of children. The study population consisted of 215 participants, comprising 119 boys and 96 girls. Briefly, the mean (SD) age of children was 9.14 (0.71) years, with the boys averaging 9.08 (0.77) and the girls 9.22 (0.64) years. The mean (SD) BMI of children was 16.6 (2.77) kg/m^2^, with the boys averaging 16.7 (2.79) and the girls 16.5 (2.75) kg/m^2^. In addition, 17.2% of the children were exposed to passive smoke. The mean (SD) vitamin A concentration of children was 0.40 (0.09) mg/L, with the boys averaging 0.41 (0.09) and the girls 0.40 (0.08) mg/L. The above variables showed no statistically significant differences between boys and girls. The levels of RTL, 5S rDNA-CN, 5.8S rDNA-CN, 18S rDNA-CN, 28S rDNA-CN, 45S rDNA-CN, and mtDNA-CN were 2.09 (0.63), 17.5 (5.34), 6.19 (2.61), 15.3 (9.39), 14.9 (5.99), 12.1 (5.20) and 137 (39.0), respectively. With the exception of 5.8S rDNA-CN, none of the other biomarkers showed differences between boys and girls.

### 3.2. Multivariable Linear Regression Analysis of Vitamin A in Relation to Genomic Biomarkers

For vitamin A with an IQR increase in concentration, the corresponding percentage changes in estimates and 95% CI of genomic biomarkers were calculated with linear regression analysis. As shown in Table 2, after adjustment for potential confounders, an IQR increase in vitamin A was significantly associated with a 9.96% increase in RTL (95% CI: 4.65%, 15.6%), a 8.00% increase in 5S rDNA-CN (95% CI: 2.84%, 13.4%), a 13.4% increase in 18S rDNA-CN (95% CI: 2.93%, 24.9%), and a 7.41% increase in 45S rDNA-CN (95% CI: 0.27%, 15.1%). The association between vitamin A and 45S rDNA-CN became non-significant after FDR correction for multiple comparisons.

In addition, for RTL, compared with Q1 of vitamin A, the estimates and 95% CI were 18.3% (7.07%, 29.4%) for Q4, with a significant trend across quartiles (*p* for trend < 0.05). For 5S rDNA-CN, compared with Q1, the estimates and 95% CI were 11.6% (0.86%, 22.4%) for Q2, 12.4% (1.72%, 23.1%) for Q3, and 21.6% (10.8%, 32.5%) for Q4, with a significant trend across quartiles (*p* for trend < 0.001). For 18S rDNA-CN, compared with Q1, the estimates and 95% CI were 32.0% (10.4%, 53.5%) for Q4, with a significant trend across quartiles (*p* for trend < 0.05). For 45S rDNA-CN, compared with Q1, the estimates and 95% CI were 20.8% (5.65%, 36.0%) for Q4, with a significant trend across quartiles (*p* for trend < 0.05).

### 3.3. Dose–Response Analysis of Vitamin A in Relation to Genomic Biomarkers

**Figure 2:**
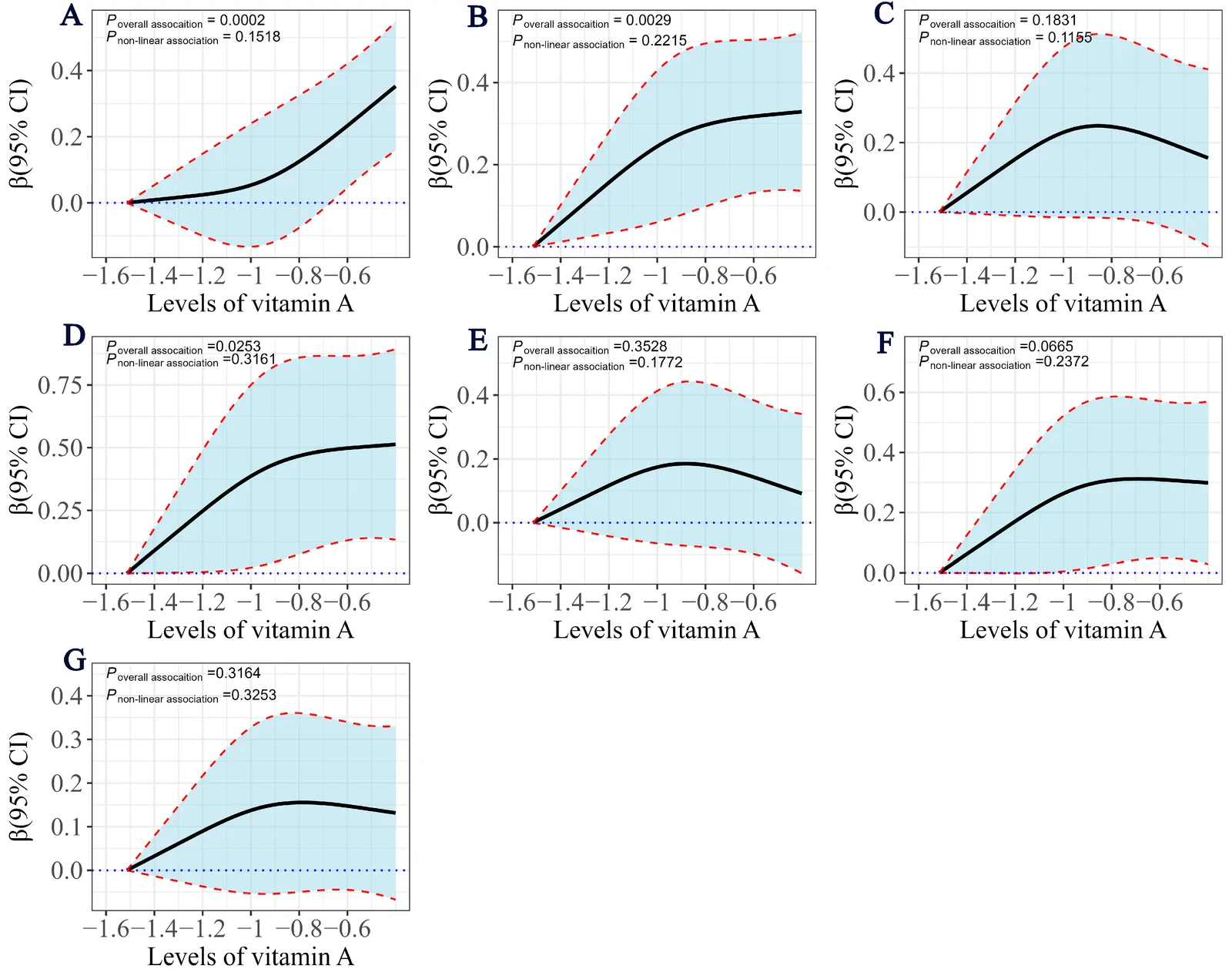
The restricted cubic spline (RCS) regression for the association between vitamin A and genomic biomarkers. (**A**): relative telomere length; (**B**): 5S ribosomal DNA copy number; (**C**): 5.8S ribosomal DNA copy number; (**D**): 18S ribosomal DNA copy number; (**E**): 28S ribosomal DNA copy number; (**F**): 45S ribosomal DNA copy number; (**G**): Mitochondrial DNA Copy Number.

The dose–response relationship of serum vitamin A levels with genomic biomarkers was examined by RCS regression (Figure 2). An overall positive association was observed between vitamin A and RTL, 5S rDNA-CN, and 18S rDNA-CN, with the estimates gradually increasing as vitamin A levels increased and no evidence of non-linearity (*p* for overall association < 0.05; *p* for non-linearity > 0.05). In addition, the association between vitamin A and 45S rDNA-CN was marginally significant in RCS regression (*p* for overall association = 0.07; *p* for non-linearity > 0.05). Consistent with the multivariable linear regression results, no significant overall associations were observed between vitamin A and 5.8S rDNA-CN, 28S rDNA-CN, and mtDNA-CN.

### 3.4. Stratified Analysis by Sex

Stratified analyses were performed to evaluate the consistency of the associations between different sexes. Interaction was estimated by including multiplicative interaction terms. As shown in Table 3, for boys, significant positive associations were identified between vitamin A and RTL. For girls, significant positive associations were identified of vitamin A with 5S rDNA-CN. The associations of vitamin A with 18S rDNA-CN and 45S rDNA-CN were significant, but they became non-significant after FDR correction for multiple comparisons. No significant differences between the sexes were observed.

## 4. Discussion

The present population-based prospective cohort study systematically explored the associations between serum vitamin A and key multi-copy genomic biomarkers (RTL, mtDNA-CN, and rDNA-CNs) among school-age children, filling a critical gap in pediatric nutritional genomics research. The core findings revealed that higher serum vitamin A levels were significantly and positively associated with elevated RTL, 5S rDNA-CN, and 18S rDNA-CN in children, with linear dose–response relationships verified by restricted cubic spline regression. These results preliminarily support our research hypothesis and provide novel epidemiological evidence linking vitamin A status to genomic biomarkers in pediatric populations.

Telomeres protect the ends of chromosomes but shorten following cell division in the absence of telomerase activity. When telomeres become critically short or damaged, a DNA damage response is activated. Telomeres then become dysfunctional and trigger cellular senescence or death [38,39]. Available research confirms telomere attrition is modifiable, given substantial heterogeneity in telomere shortening rates independent of chronological age [32]. Epidemiological evidence has validated that telomere length is strongly modulated by amendable lifestyle determinants, among which nutritional status acts as a critical regulatory factor [40,41]. Consistently, antioxidant vitamins, including carotene as the precursor of vitamin A, have been repeatedly associated with preserved longer telomere length in existing studies [32]. In line with these established conclusions regarding nutritional regulation on telomere homeostasis, our current study observed a positive correlation between vitamin A levels and RTL. This finding further supplements and corroborates prior relevant reports focusing on the nutritional regulation of telomere maintenance.

Like telomeres, preservation of rDNA loci consisting of the highly repetitive rRNA genes represents one of the most critical aspects of genome maintenance [12]. Lower rDNA-CN has consistently been associated with higher body weight and body mass index [26]. In the present pediatric cohort, we observed positive associations between vitamin A and 5S and 18S rDNA-CN. In this context, sufficient vitamin A may serve as a nutritional factor that stabilizes rDNA repeats and prevents rDNA attrition.

Mitochondrial dysfunction, a hallmark of the aging process, could disrupt energy homeostasis, and a reduction in mtDNA-CN is believed to play a critical role in the initiation and progression of atherosclerosis [10,42,43]. Despite the well-established mitochondrial protective effects of the active metabolite of vitamin A via suppressing endoplasmic reticulum stress and activating the ferroptosis signaling pathway [44], the present study failed to observe a statistically significant association between circulating vitamin A levels and peripheral blood mtDNA-CN in our study population.

As a pivotal fat-soluble micronutrient, vitamin A participates in a wide range of physiological processes, with its biological functions predominantly mediated by its active metabolite, RA [45]. RA binds to nuclear retinoid receptors (RAR/RXR) to initiate downstream transcriptional regulation, thereby exerting potent antioxidant, anti-inflammatory, and anti-senescence biological effects [18]. Excessive reactive oxygen species (ROS) accumulation and persistent inflammatory activation are core upstream drivers of altered telomere homeostasis [46], mitochondrial dysfunction [47], and rDNA copy number perturbation [48]. Accumulating evidence has demonstrated that RA effectively suppresses endogenous ROS overproduction, modulates the nuclear factor erythroid 2-related factor 2-Kelch-like ECH-associated protein 1 (Nrf2-Keap1) antioxidant signaling pathway, and alleviates chronic inflammatory stress [18], which are critical for preserving genomic integrity and stabilizing repetitive genomic sequences. In addition, the active metabolite of vitamin A, RA, recruits histone-modifying complexes via RAR-RXR nuclear receptors to remodel chromatin status and regulate the transcription of DNA-repair-related genes, thereby maintaining genomic stability. Meanwhile, the provitamin A β-carotene modulates DNA methylation and miRNA expression, exerting anti-genotoxic effects synergistically at the epigenetic level [19]. At physiological appropriate doses, RA exerts genome-protective effects, whereas excessive supplementation induces pro-oxidant effects [19]. Other carotenoids (astaxanthin, zeaxanthin, and lutein) also exert antigenotoxic effects. Astaxanthin could block oxidative stress-induced DNA damage by preventing Ku protein degradation and inhibiting apoptosis [49]. Lutein could inhibit oxidative stress-induced cell apoptosis, and zeaxanthin could alleviate retinal oxidative stress and inflammatory damage [50]. Other natural antioxidant compounds, including polyphenols (e.g., epigallocatechin-3-gallate, quercetin, resveratrol), have also demonstrated potential in reducing oxidative stress [50]. In addition, vitamin E (α-tocopherol), a fat-soluble vitamin, protects biological membranes from ROS-mediated lipid peroxidation [51]. According to the results of in-vitro experiments, antioxidant capacity is as follows: α-tocopherol > β-carotene > zeaxanthin > lutein > astaxanthin [52].

This study has several notable strengths that enhance the scientific rigor and clinical value of the findings. First, to our knowledge, it is the first study to systematically elaborate the independent prospective associations between vitamin A nutritional level and the multi-copy genomic biomarkers among children, who are in a critical developmental window with high genomic susceptibility. Second, the study employed a population-based prospective cohort design, which overcomes the inherent limitations of cross-sectional studies. Third, the study adopted a rigorous methodological design and standardized detection protocols. Serum vitamin A was quantitatively measured by high-precision liquid chromatography-tandem mass spectrometry, and genomic biomarkers were detected by standardized real-time qPCR with strict DNA quality control and triplicate sample detection to ensure accurate and reliable experimental data. However, there are a few limitations. First, the single-center design limits the generalizability of the findings. All participants were recruited from a single regional sample source without multi-center population verification. Second, the study has only conducted one follow-up assessment of genomic biomarkers, failing to capture the dynamic continuous changes in vitamin A levels and genomic biomarkers during children’s growth. Long-term continuous dynamic monitoring is lacking, and causal associations cannot be established. Third, although common confounding factors such as demographic characteristics and passive smoking were adjusted for, other factors affecting genomic stability and vitamin A metabolism, including dietary structure, nutritional supplements, socioeconomic status, physical activity level, inflammation, pubertal status, environmental exposures, and other micronutrient levels, were not included in the adjustment model, which may have an important impact on the accuracy of the correlation results.

These findings enrich the evidence base for nutritional regulation of repetitive genomic elements in children and carry potential public health implications for nutritional monitoring and genome protection in early life. Future studies should incorporate repeated longitudinal measurements of vitamin A and multi-copy genomic biomarkers, comprehensively adjust for coexisting dietary and micronutrient exposures, and perform mechanistic experiments to elucidate the biological pathways through which vitamin A maintains telomere and rDNA homeostasis.

## 5. Conclusions

This prospective cohort study systematically examined the associations between serum vitamin A concentrations and multi-copy genomic biomarkers in school-age children. Telomere length and rDNA copy number are sensitive markers of genomic integrity and biological aging in children. Our primary findings showed that baseline serum vitamin A concentrations were positively and linearly associated with children’s RTL, as well as 5S and 18S rDNA copy numbers at the four-year follow-up. Most prior studies were cross-sectional and unable to establish temporality between vitamin A and genomic biomarkers. This study fills a gap in prospective epidemiological evidence for pediatric nutritional genomics. However, these findings should be interpreted cautiously, as only baseline serum vitamin A was measured and other nutritional co-factors were not fully considered. Longitudinal replication studies are warranted to verify this association and explore underlying mechanisms.

## Figures and Tables

**Table 1:** Characteristics of the study participants (*N* = 215).

Variables	Overall (*N* = 215)	Boys (*N* = 119)	Girls (*N* = 96)	*p*-Values
Age (years), mean (SD)	9.14 (0.71)	9.08 (0.77)	9.22 (0.64)	0.24
BMI (kg/m^2^)	16.6 (2.77)	16.7 (2.79)	16.5 (2.75)	0.67
Passive smoke				0.86
Yes	17.2%	20 (16.8%)	17 (17.7%)	
No	82.8%	99 (83.2%)	79 (82.3%)	
Vitamin A (mg/L), mean (SD)	0.40 (0.09)	0.41 (0.09)	0.40 (0.08)	0.32
RTL, mean (SD)	2.09 (0.63)	2.04 (0.59)	2.16 (0.68)	0.28
5S rDNA-CN, mean (SD)	17.5 (5.34)	17.2 (5.48)	17.8 (5.16)	0.21
5.8S rDNA-CN, mean (SD)	6.19 (2.61)	6.45 (2.47)	5.88 (2.75)	0.01
18S rDNA-CN, mean (SD)	15.3 (9.39)	16.4 (10.8)	13.9 (7.07)	0.29
28S rDNA-CN, mean (SD)	14.9 (5.99)	15.2 (5.57)	14.6 (6.48)	0.09
45S rDNA-CN, mean (SD)	12.1 (5.20)	12.7 (5.64)	11.5 (4.53)	0.12
MtDNA-CN, mean (SD)	137 (39.0)	140 (40.3)	134 (37.3)	0.49

Abbreviations: SD: standard deviation; BMI: body mass index; RTL: relative telomere length; rDNA-CN: ribosomal DNA copy number; mtDNA-CN: mitochondrial DNA copy number; *p*-values are for the Mann–Whitney U test across the different sexes of each characteristic.

**Table 2:** Associations of serum vitamin A and genomic biomarkers using multiple linear regression models.

	%Δ (95% CI)						
	RTL	5S rDNA-CN	5.8S rDNA-CN	18S rDNA-CN	28S rDNA-CN	45S rDNA-CN	MtDNA-CN
Serum vitamin A per IQR increment	**9.96** **(4.65, 15.6)**	**8.00** **(2.84, 13.4)**	3.39 (−3.13, 10.4)	**13.4** **(2.93, 24.9)**	1.77 (−4.48, 8.43)	**7.41** **(0.27, 15.1)**	2.79 (−2.28, 8.12)
FDR-adjusted *p*-value	**0.001**	**0.008**	0.367	**0.026**	0.586	**0.073**	0.399
Serum vitamin A quartile							
Q1 (<0.34 mg/L)	Reference	Reference	Reference	Reference	Reference	Reference	Reference
Q2 (0.34 to <0.39)	6.09 (−4.99, 17.2)	**11.6** **(0.86, 22.4)**	12.6 (−1.51, 26.8)	19.1 (−2.16, 40.5)	6.45 (−7.42, 20.3)	12.7 (−2.36, 27.7)	10.8 (−0.26, 21.9)
Q3 (0.39 to <0.45)	3.96 (−7.04, 14.9)	**12.4** **(1.72, 23.1)**	−3.15 (−17.2, 10.9)	5.90 (−15.3, 27.0)	−7.99 (−21.8, 5.77)	−1.16 (−16.1, 13.8)	−0.09 (−11.1, 10.9)
Q4 (≥0.45)	**18.3** **(7.07, 29.4)**	**21.6** **(10.8, 32.5)**	**17.1** **(2.78, 31.4)**	**32.0** **(10.4, 53.5)**	8.96 (−5.05, 23.0)	**20.8** **(5.65, 36.0)**	9.62 (−1.59, 20.8)
*p* for trend	0.002	<0.001	0.088	0.013	0.423	0.030	0.327

Bold values indicate statistical significance. RTL: relative telomere length; rDNA-CN: ribosomal DNA copy number; mtDNA-CN: mitochondrial DNA copy number.

**Table 3:** Associations of serum vitamin A and genomic biomarkers stratified by sex.

Genomic Biomarkers		%Δ (95% CI)			
	*p* for Interaction	Boys (*N* = 119)	FDR-Adjusted *p*-Value	Girls (*N* = 96)	FDR-Adjusted *p*-Value
RTL	0.607	13.5 (6.38, 21.0)	0.001	7.91 (−0.30, 16.8)	0.104
5S rDNA-CN	0.446	6.61 (−0.49, 14.2)	0.239	10.2 (2.42, 18.6)	0.035
5.8S rDNA-CN	0.934	3.10 (−5.02, 11.9)	0.648	3.10 (−7.45, 14.9)	0.575
18S rDNA-CN	0.394	8.78 (−5.46, 25.2)	0.554	20.1 (4.93, 37.4)	0.059
28S rDNA-CN	0.433	−0.04 (−7.67, 8.22)	0.992	4.69 (−5.68, 16.2)	0.539
45S rDNA-CN	0.372	4.40 (−5.18, 14.9)	0.661	11.7 (1.06, 23.4)	0.071
mtDNA-CN	0.769	1.42 (−5.16, 8.46)	0.790	3.35 (−4.69, 12.1)	0.491

## Data Availability

Supporting data can be provided by the corresponding authors upon reasonable request.

## Supplementary Materials

The following supporting information can be downloaded at https://www.mdpi.com/article/10.3390/nu18203313/s1, Table S1: Strengthening the Reporting of Observational Studies in Epidemiology (STROBE) checklist for this prospective observational cohort study. Table S2: Mobile phase for vitamin A. Table S3: The primers for ribosomal DNA copy number. Table S4: The primers for mitochondrial DNA copy number. Table S5: The primers for relative telomere length.

## Abbreviations

The following abbreviations are used in this manuscript:

RTL	Relative telomere length
RA	Retinoic acid
rDNA-CN	Ribosomal DNA copy number
mtDNA-CN	Mitochondrial DNA copy number
SD	Standard deviation
BMI	Body mass index
RCS	Restricted cubic spline
