# Supplementary material for: Serum Vitamin A in Relation to Genomic Biomarkers in Children: A Prospective Study
Source: Nutrients. 2026 Oct 9;18(20):3313. doi: 10.3390/nu18203313 (PMC13708395; doi:10.3390/nu18203313)
Supplement: Supplementary file 1 [file nutrients-18-03313-s001.zip › nutrients-4509159-supplementary.pdf]

Serum Vitamin A in Relation to Genomic Biomarkers in Children: A Prospective Study

Yan Wang[1†], Kuiqing Huang[2†], Chen Liu[3], Yi Wang[3], Jinghan Jiang[2], Zhijian Chen[1], Ronghua Zhang[1,3], Peiwei Xu[1,3*], Xiaoming Lou[1,3]

[1]Zhejiang Provincial Center for Disease Control and Prevention, Hangzhou 310051, PR China.

[2]Yiwu Center for Disease Control and Prevention, Yiwu 322000, Zhejiang, PR China

[3]School of Public Health, Hangzhou Medical College, Hangzhou 310059, PR China

† Co-first author

* Corresponding author: Zhejiang Provincial Center for Disease Control and Prevention, Hangzhou 310051, PR China, E-mail address: pwxu@cdc.zj.cn (Peiwei Xu);

Methods

2.3. Measurement of vitamin A

Serum vitamin A concentrations were quantified using an ultra-high-performance liquid chromatography–tandem mass spectrometry (UPLC-MS/MS) system (TRIPLE QUAD 4500, AB SCIEX, USA). All analytical standards, including vitamin A and its stable isotope internal standard (vitamin A-d5), were purchased from Toronto Research Chemicals (TRC, Canada). Chromatographic-grade methanol, ethanol, formic acid, and n-hexane were obtained from CNW Technologies (Shanghai, China), and 2,6-di-tert-butyl-4-methylphenol (BHT) was purchased from Tokyo Chemical Industry Co., Ltd. (TCI, Shanghai, China) to prevent lipid oxidation during sample pretreatment.

For serum sample pretreatment, 200 μL serum was transferred into a centrifuge tube, followed by the addition of 400 μL ethanol solution containing internal standard and 0.1 mg/mL BHT. The mixture was vortexed for 30 s, and 1 mL n-hexane was then added for further extraction with continuous vortexing for 10 min. The mixture was centrifuged at 13000 rpm for 5 min at 4 °C. A total of 850 μL of the upper organic phase was collected and vacuum freeze-dried. The dried residue was reconstituted with 100 μL ethanol, vortexed for 5 min, and centrifuged again at 13000 rpm for 5 min at 4 °C. Subsequently, 90 μL of the supernatant was transferred to an injection vial for instrumental analysis.

Chromatographic separation was performed on a Kinetex® 2.6 μm C18 column (3 × 30 mm). Mobile phase A was 0.1% formic acid in water, and mobile phase B was 0.1% formic acid in methanol. The injection volume was 10 μL, the flow rate was 0.7 mL/min, and the column temperature was maintained at 40 °C. The gradient elution program is shown in Supplementary Table S2.

Mass spectrometric detection was performed in positive electrospray ionization (ESI+) mode. The ion spray voltage was set at 5500 V and the source temperature was set at 450 °C.

2.4. Measurement of genomic biomarkers

Genomic DNA was extracted using a HiPure Blood DNA Mini Kit (D3111, Magen Biotechnology, Guangzhou, China). Briefly, the sample was transferred into a 1.5 mL centrifuge tube, mixed with 220 μL of Buffer ATL and 20 μL of Proteinase K by vortexing, and incubated at 55 °C for 1–3 h until complete digestion and clarification with several inversions during incubation. Then, 10 μL of RNase A was added to the digest, mixed by inversion, and kept at room temperature for 10 min. Subsequently, 250 μL of Buffer DL was added, followed by high-speed vortexing for 10 s and incubation in a 70 °C water bath for 10 min. After centrifugation at 12,000 × g for 3 min, the supernatant was transferred to a new 1.5 mL centrifuge tube, mixed with 250 μL of absolute ethanol by vortexing for 15 s. The mixture including precipitate was loaded onto the spin column assembled in a 2 mL collection tube and centrifuged at 12,000 × g for 1 min; the sample was loaded in portions if the volume exceeded 750 μL, and 14,000 × g centrifugation for 3–5 min was applied when column clogging occurred. The flow-through was discarded, and the column was washed sequentially with 500 μL of Buffer GW1 and 650 μL of Buffer GW2, each followed by centrifugation at 12,000 × g for 1 min. After the final wash, the column was centrifuged at 12,000 × g for 2 min to remove residual liquid. The column was transferred to a new 1.5 mL centrifuge tube, 70 μL of Buffer AE preheated to 70 °C was added onto the center of the column membrane, and the tube was kept stationary for 3 min before centrifugation at 12,000 × g for 1 min. Finally, the DNA binding column was discarded; the obtained DNA was stored at 2–8 °C for short-term use and at −20 °C for long-term preservation. DNA integrity was examined via 1% gel electrophoresis, and DNA purity was determined using a Microdrop SE spectrophotometer (BIO-DL, Shanghai, China), with acceptable OD260/280 values between 1.6 and 2.0.

Quantitative real-time PCR (qPCR) was performed to detect target genes using an ABI ViiA 7 real-time PCR system (Applied Biosystems, Waltham, MA, USA). Briefly, the SYBR Green Master Mix (TSE203, TSINGKE, Beijing, China) was thawed at 4 °C, mixed gently by inversion, and briefly centrifuged. A 20 μL reaction system was prepared on ice, containing 10 μL of the SYBR Green Master Mix, 0.4 μL of the forward primer (10 μM), 0.4 μL of the reverse primer (10 μM), and 20 ng of template

DNA, with RNase-free water added to a final volume of 20 μL. The reaction mixture was briefly centrifuged to ensure all liquid was collected at the bottom of the wells. The amplification procedure was set as follows: initial predenaturation at 95 °C for 5 min, followed by 40 cycles of 95 °C for 10 s and 60 °C for 30 s. Fluorescence signals were collected at the end of each cycle.

Table S1. Strengthening the Reporting of Observational Studies in Epidemiology (STROBE) checklist for this prospective observational cohort study.

Section and topic	Item No.	STROBE recommendation	Reported on Page / Line
Title and abstract	1	Indicate the study's design with a commonly used term in the title or the abstract; provide in the abstract an informative and balanced summary of what was done and what was found	Line 20-25; Line 25-34
Introduction			
Background/rationale	2	Explain the scientific background and rationale for the investigation being reported	Line 39-72
Objectives	3	State specific objectives, including any prespecified hypotheses	Line 106-121
Methods			
Study design	4	Present key elements of study design early in the paper	Line 127-130
Setting	5	Describe the setting, locations, and relevant dates, including periods of recruitment, exposure, follow-up, and data collection	Line 125-139
Participants	6	(a) Cohort: Give eligibility criteria, sources and selection methods of participants; describe methods of follow-up.	Line 130-137
Variables	7	Clearly define outcomes, exposures, predictors, confounders, effect modifiers; diagnostic criteria if applicable	Line 148-176
Data sources/measurement	8*	For each variable, give data sources and measurement methods; (If there are multiple groups, the comparability of the determination methods among the groups should also be described.)	Line 148-226
Bias	9	Describe any efforts to address potential sources of bias	Line 148-154
Study size	10	Explain how the study size was determined	Line 126-139
Quantitative variables	11	Explain how quantitative variables were handled in analyses; describe groupings and rationale if used	Line 228-232

Section and topic	Item No.	STROBE recommendation	Reported on Page / Line
Statistical methods	12	(a) Describe all statistical methods, including those used to control for confounding.(b) Describe any methods used to examine subgroups and interactions.(c) Explain how missing data were addressed.(d) If applicable, describe any sensitivity analyses.	Line 233-243
Results			
Participants	13*	(a) Report numbers of individuals at each stage of study recruitment, eligibility, included in follow-up, included in analysis.(b) Describe reasons for non-participation or loss to follow-up.(c) Report follow-up duration.	Line 263
Descriptive data	14*	(a) Give baseline demographic and clinical characteristics of participants.(b) Indicate number of participants with missing data for each variable of interest.	Line 263-273
Outcome data	15*	Report numbers of outcome events or summary measures over time	Line 280-296
Main results	16	(a) Give unadjusted and adjusted estimates with precision (e.g., 95%CI). State which confounders were adjusted for and why they were included.(b) Report category boundaries when continuous variables were categorized.(c) If relevant, consider translating estimates into absolute risk at a relevant time point.	Line 282-296
Other analyses	17	Report other analyses done, e.g., subgroup or interaction analyses, sensitivity analyses	Line 317-324
Discussion			
Key results	18	Summarize key results with reference to study objectives	Line 331-339
Limitations	19	Discuss limitations of the study, addressing sources of potential bias, imprecision and, if relevant, multiplicity of analyses	Line 412-423
Interpretation	20	Provide a cautious overall interpretation considering objectives, limitations, multiplicity of analyses, results from similar studies, and other relevant evidence	Line 445-455

**Section and topic**	**Item No.**	**STROBE recommendation**	**Reported on Page / Line**
Generalisability	21	Discuss the external validity (generalizability) of the findings	Line 412-414
Other information			
Funding	22	Give the source of funding and the role of the funders for the present study	Line 461-466

Table S2. Mobile phase for vitamin A.

**Time（min）**	**A（%）**	**B（%）**
**0**	60	40
**0.4**	60	40
**1.2**	25	75
**3**	0	100
**4.5**	0	100
**4.51**	60	40
**5.0**	60	40

Table S3. The primers for ribosomal DNA copy number.

Primer	Sequence（5'-3'）
**28S-F**	AGCCGACTTAGAACTGGTGC
**28S-R**	AGTTACTCCCGCCGTTTACC
**18S-F**	GCGGCTTGGTGACTCTAGAT
**18S-R**	CGTTTCTCAGGCTCCCTCTC
**5.8S-F**	GTGGATCACTCGGCTCGTG
**5.8S-R**	GCAAGTGCGTTCGAAGTGTC
**5S-F**	CCATACCACCCTGAACGCG
**5S-R**	CTACAGCACCCGGTATTCCC
**TP53-F**	CCTGTGCAGTTGTGGGTCA
**TP53-R**	TACCATCACCATCGGAGCAG

Table S4. The primers for mitochondrial DNA copy number.

Primer	Sequence（5'-3'）
**ND1-F**	AATCGCCATAGCCTTCCTAACAT
**ND1-R**	GGCGTCTGCAAATGGTTGTAA
**H19-F**	GTACCCACCTGTCGTCC
**H19-R**	GTCCACGAGACCAATGACTG

Table S5. The primers for relative telomere length.

Primer	Sequence（5'-3'）
**telomere-F**	ACACTAAGGTTTGGGTTTGGGTTTGGGTTTGGGTTAGTGT
**telomere-R**	TGTTAGGTATCCCTATCCCTATCCCTATCCCTATCCCTAACA
**36B4-F**	ACTGGTCTAGGACCCGAGAAG
**36B4-R**	TCAATGGTGCCTCTGGAGATT
